# Ultraviolet (IUV) and mass spectrometry (IMS) imaging for the deconvolution of microbial interactions

**DOI:** 10.1186/s12918-018-0617-3

**Published:** 2018-11-20

**Authors:** Víctor González-Menéndez, Germán Martínez, Rachel Serrano, Francisca Muñoz, Jesús Martín, Olga Genilloud, José R. Tormo

**Affiliations:** Fundación MEDINA, Avda Conocimiento 34, CDFA, PTS, 18016 Granada, Spain

**Keywords:** IUV, IMS, Metabolomics, Co-culturing, Microbial interactions

## Abstract

**Background:**

Spatial localization of natural products or proteins during microbial interactions can help to identify new antimicrobials both as offensive or defensive agents. Visible spatial interactions have been used for decades to enhance Drug Discovery processes both in industry and academia.

**Results:**

Herein we describe an automated micro-extraction methodology, that coupled with the previously described HPLC-Studio 2.0 software and the new development, the MASS-Studio 1.0 software, can combine multiple chemical analyses to generate ultraviolet (UV) and mass spectrometry (MS) images from traditional affordable analytical equipment. As a proof of concept, we applied this methodology on two microbial antagonisms observed among co-habitant endophytes isolated from endemic plants of arid areas of the south of Europe.

**Conclusions:**

The use of UV and MS images highlighted interacting naturals products and allowed clear identification of induced molecules of interest not produced by the strains when cultured individually.

## Background

Drug Discovery for new chemical entities and innovative compound design relies on natural products for more than half of the drugs in development [1]. So far more than 42% of known bioactive compounds have been described as produced by filamenting fungi and many of these molecules with pharmacological applications were developed for clinical uses, especially as antibiotics and antifungals among other applications [2, 3]. However, a general perception is that the emerging rates of discovery of new molecules, especially new antibiotics, are decreasing after half a century of continued research on fungal diversity and axenic fermentation-based processes [4].

Fungal genome mining has revealed the high number of gene clusters involved in the biosynthesis of fungal secondary metabolites (SMs) that are not produced in strains cultivated axenically in laboratory conditions [5, 6]. Recently, several approaches have been applied to foster the expression of these unexpressed pathways and to promote their biosynthesis. These strategies have included: the manipulation of medium and growth conditions in miniaturized nutritional arrays [7], the application of transformation techniques for the generation of gene knockouts, the exchange of native gene promoters with constitutive or inducible promoters or the overexpression of transcription factors [8], or the co-cultivation of more than one microorganism in constant interaction [9].

Traditional screening processes to discover new bioactive molecules, involve culturing a single microbial strain, however the use of co-cultures presents new opportunities for the activation of cryptic biosynthetic pathways. Microorganisms can present an antagonistic reaction in presence of other microorganisms that promotes changes in their morphology and the production of SMs, enzymes and other compounds in the interaction zone [10].

Co-culturing has proved to be an effective tool to simulate the physiological conditions that occur during microbial interaction in their natural environment and may have an enormous potential for the discovery of new molecules with therapeutic approaches [11–13].

Recent detection techniques try to evaluate natural products by Image Mass Spectrometry (IMS) [14, 15] by remarking its high level of suitability for analyzing microbial interactions and detecting the activation of cryptic pathways [16, 17]. Unfortunately, most of these techniques rely on the use of very expensive or innovative ionization heads in the Mass Spectrometer (MS) (ie MALDI, or nano-DESI respectively). Most of the common MS for natural products are electrospray units that cover mass ranges from 150 to 1500 Da, more suitable for natural products extracts. Recently, we have developed in our lab the MASS-Studio 1.0 software tool for high-throughput analysis of batches of samples analyzed by LC-ESI-MS equipment. Herein we decided to combine miniaturized chemical extractions with HPLC-Studio 2.0 [18, 19] and MASS-Studio 1.0 [20] utilities to generate ultraviolet and mass images from LC-ESI-MS analyses.

## Methods

Endophytic strains were isolated as described previously by Gonzalez-Menendez et al. (2016) [19]. Strains interaction were performed by co-culturing on malt agar (malt extract Difco™ 20 g, agar 20 g and 1000 mL deionized H_2_O) for 14 days at 22 °C and 70% of relative humidity, and said agar of the positive antagonist was separated into 80 portions corresponding to 80 microplate wells. All co-culture portions were extracted with acetone, shaking at 220 rpm for 1 h and the samples were dried in a Genevac HT-8. Finally, the dried samples were suspended in 500 μl of 20% DMSO. The samples were analyzed by UPLC-UV and by low resolution mass spectrometry (LR-MS) in the range of positive m/z for each extract. Mass ion detection was performed in a ramp from 150 *m*/*z* to 1500 *m*/*z* in positive and negative modes. MASS-Studio 1.0 software was used for generating the mass spectrometry imaging for each co-culture. Comparison with proprietary database of more than 950 known microbial standards was performed by low resolution (LC-LRMS) using the same raw data that generated the images.

Once chemical evaluation had been performed raw data corresponding to each individual analysis was recorded in ‘cvs’ files by the equipment software and HPLC-Studio 2.0 [18, 19] and MASS-Studio 1.0 [20] were used to combine all individual analyses from ultraviolet or mass spectrometry detection respectively. Typical runs in these studies compare components detected in the samples and identify if they correspond to the same metabolite or not, by bucketing in the time dimension in the case of the HPLC-Studio 2.0 or in the mass direction in the MASS-Studio 1.0 software.

**Fig. 1:**
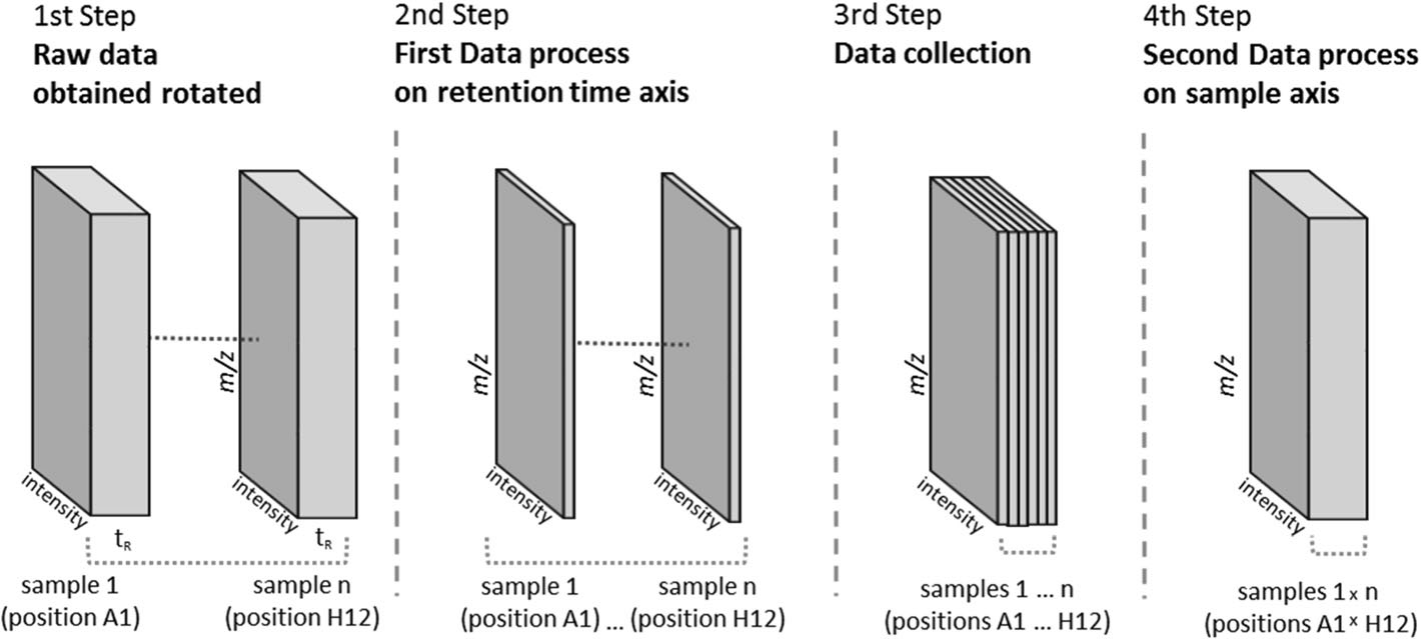
Data management in MASS-Studio 1.0 where components are processed twice

To obtain the image data management, a second reprocess was used where the spatial dimension was included and components were bucketed for all samples from the same microbial interaction (Fig. 1). In this sense all samples from each batch, including every sample for every spatial position for axenic cultures and cocultures were compared within the same data matrix for each set of strains. As a result, the combined matrix could be pivoted and represented not as quantity for each component for each sample, but as quantity of each component for each position within each batch of strains (with and without the interaction). Spatial distribution of each component was then plotted by using the Excel® commercial software application (Microsoft Corp.) [20].

Traditional analytical methods have allowed the detection of changes in the metabolite profiles that vary depending on the interacting fungi [21, 22]. Different co-culturing techniques have been developed for this purpose including liquid and solid media, but all approaches consist on culturing two or more microorganisms in a single confined environment to facilitate interactions and induce further chemical diversity [23, 24].

An automated method based on image mass spectrometry (IMS) has been used for evaluating the presence of different secondary metabolites when a clear antagonistic effect was observed in a fungal co-culture. This approach aims to analyze the microbial interactions in terms of the natural products generated (UV and MS) to evaluate the effect of antagonism and detect molecules that could be produced as a consequence, among others, of communication, attack or defense between both microorganisms. As, some of these molecules may not be produced when these microorganisms are grown axenically, there is a high probability that some of them may show biological activities with possible therapeutic purposes.

**Fig. 2:**
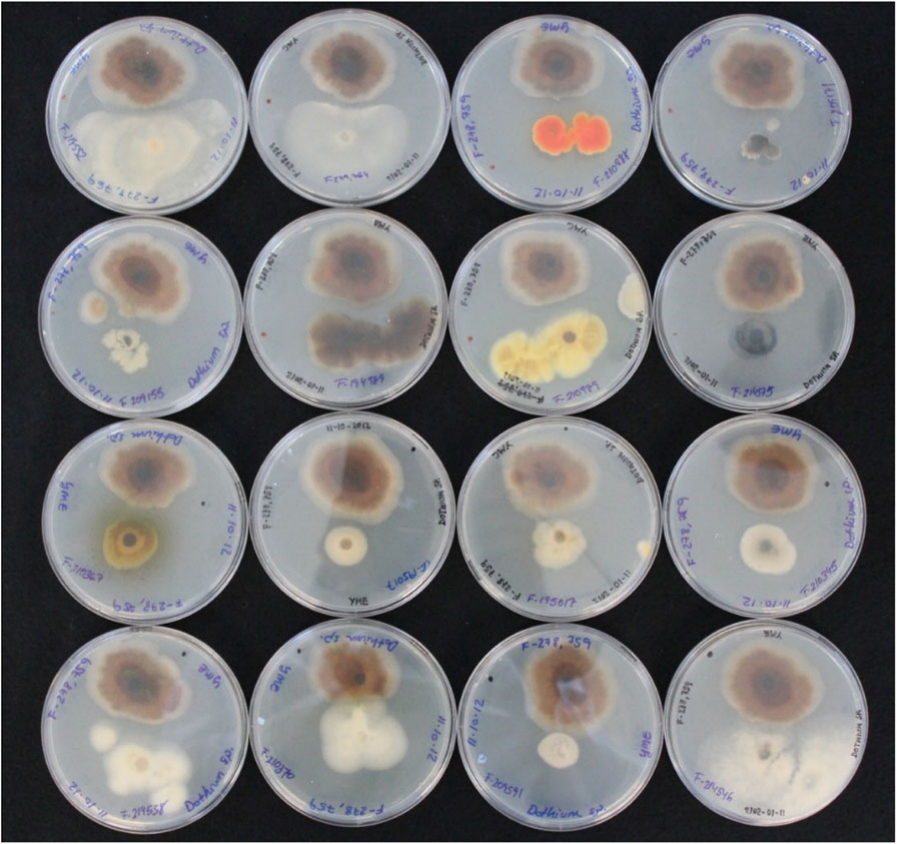
Examples of antagonisms of several endophytes co-cultures on malt agar plates

The antagonistic activity of several endophytes isolated from arid plants of Andalucía was evaluated (Fig. 2). A clear antagonistic effect was observed for two endophyte isolates from the plant *Retama sphaerocarpa*, the host-specific fungi *Dothiora* sp. CF-279759 and *Bacillus* sp. CB-293420, against the semi-parasite *Hypoxylon mediterraneum* CF-279764, isolated also from this plant.

**Fig. 3:**
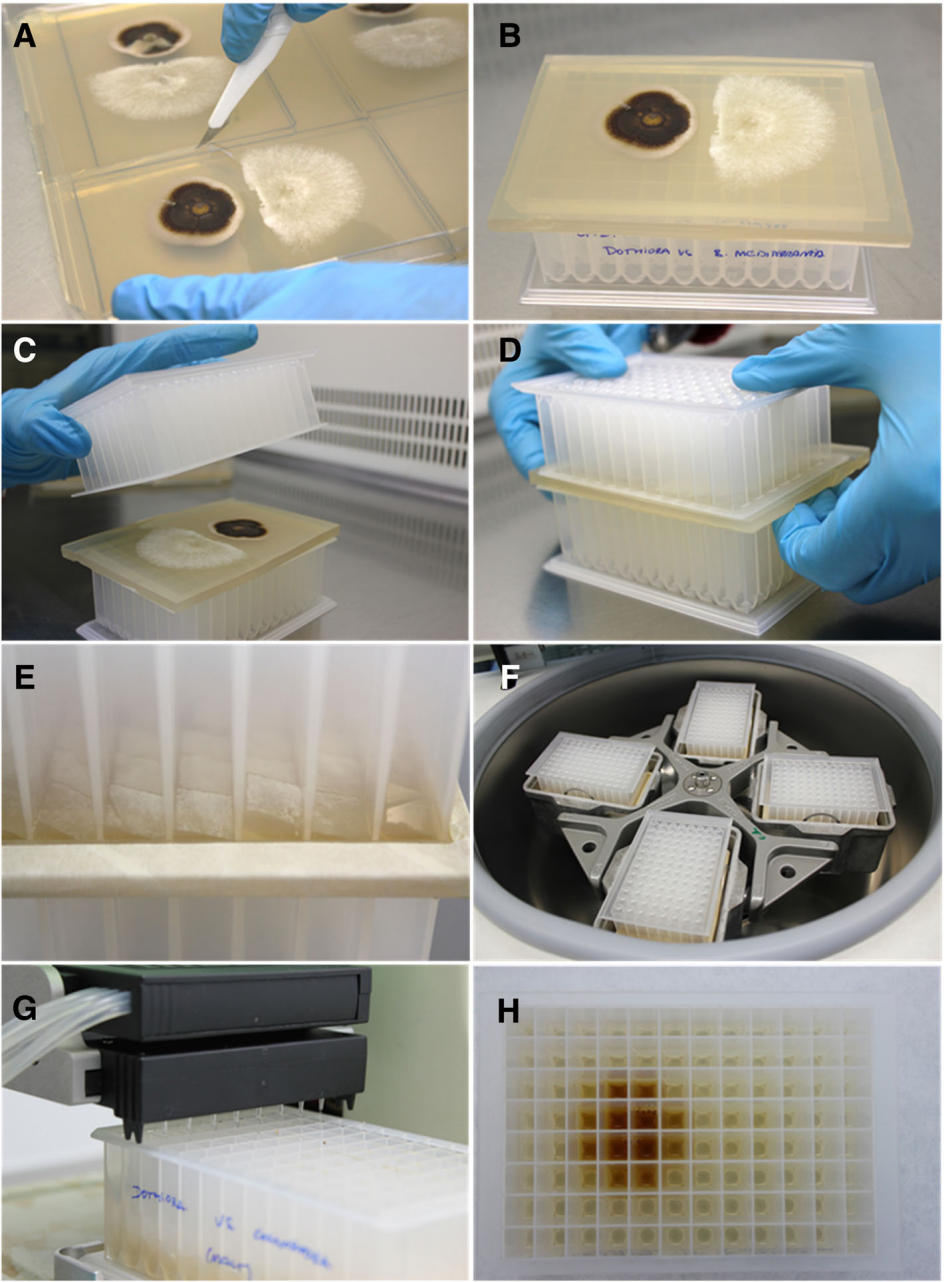
Sample management methodology performed for creating analytical extracts form microbial interactions chemical analyses. Specific steps (**a-h**) described in the text

Agar co-cultures of these two strains interacting were selected for proof of concept (POC) for image UPLC-UV and UPLC-MS spatial metabolomics analyses. After co-culturing them per triplicate on malt agar plates, sections of the same size as the destination Thermo™ ABgene® 2.2 mL AB-0661 storage plate were cut (Fig. 3a-b). By superimposing, another plate was pressed out until the co-culture was punched out into 96 square portions (Fig. 3c-e). The next step was a centrifugation at 3000 rpm for 10 min so that the portions were deposited on the bottom of the extraction plate (Fig. 3f) for later addition of acetone (Fig. 3g). After evaporation of organic solvent, the samples were resuspended in 500 μl of DMSO 20% (Fig. 3h) and 3000 rpm 10 min supernatants were analyzed by UPLC-UV and MS.

## Results and discussion

**Fig. 4:**
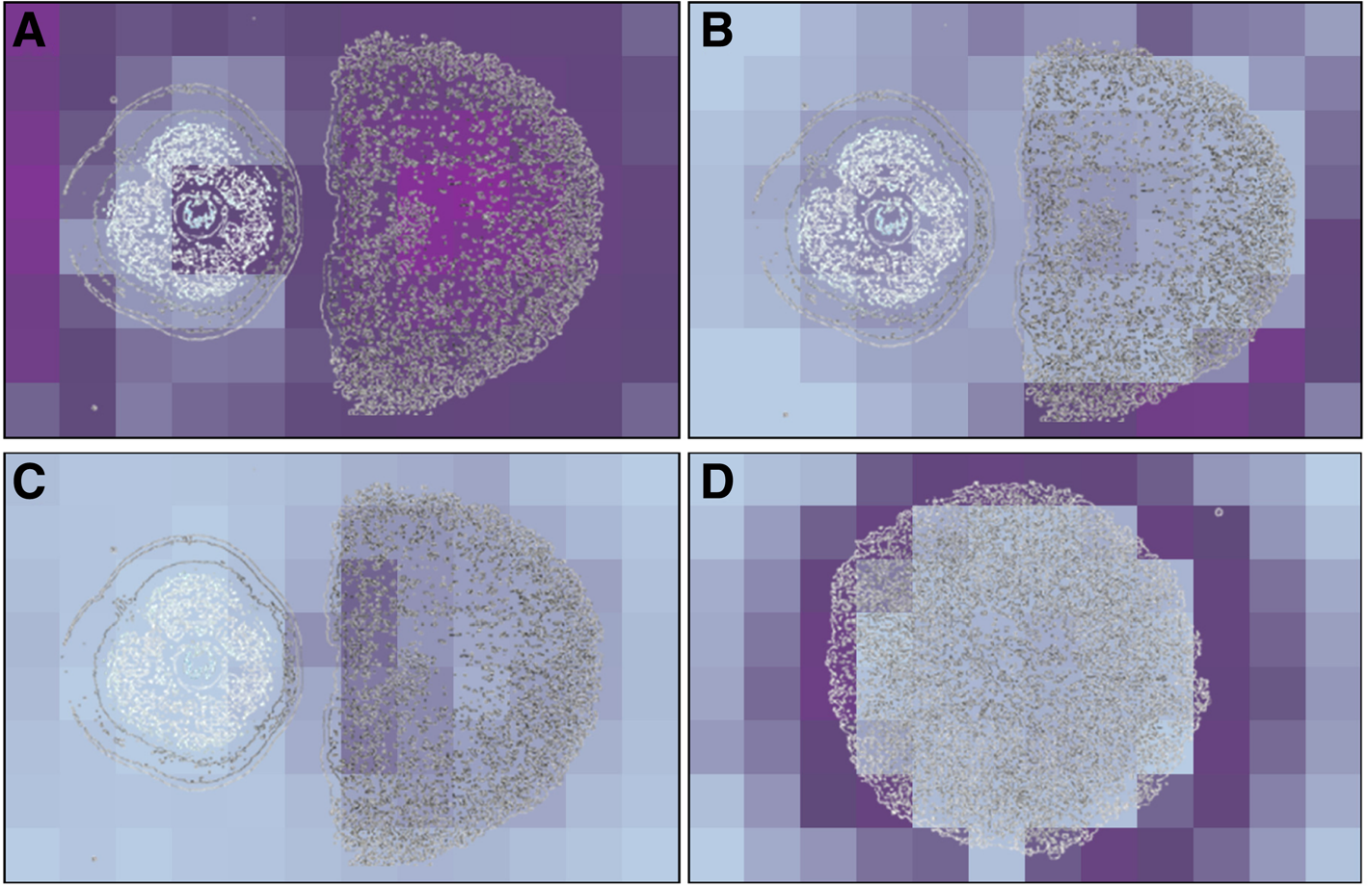
Image of microbial interactions by processing LC-UV raw data with HPLC-Studio 2.0 tool for four (**a**-**d**) UV components at 3.57, 4.90, 4.16 and 4.50 min of UHPLC retention times, respectively, that presented differential spatial distributions

**Fig. 5:**
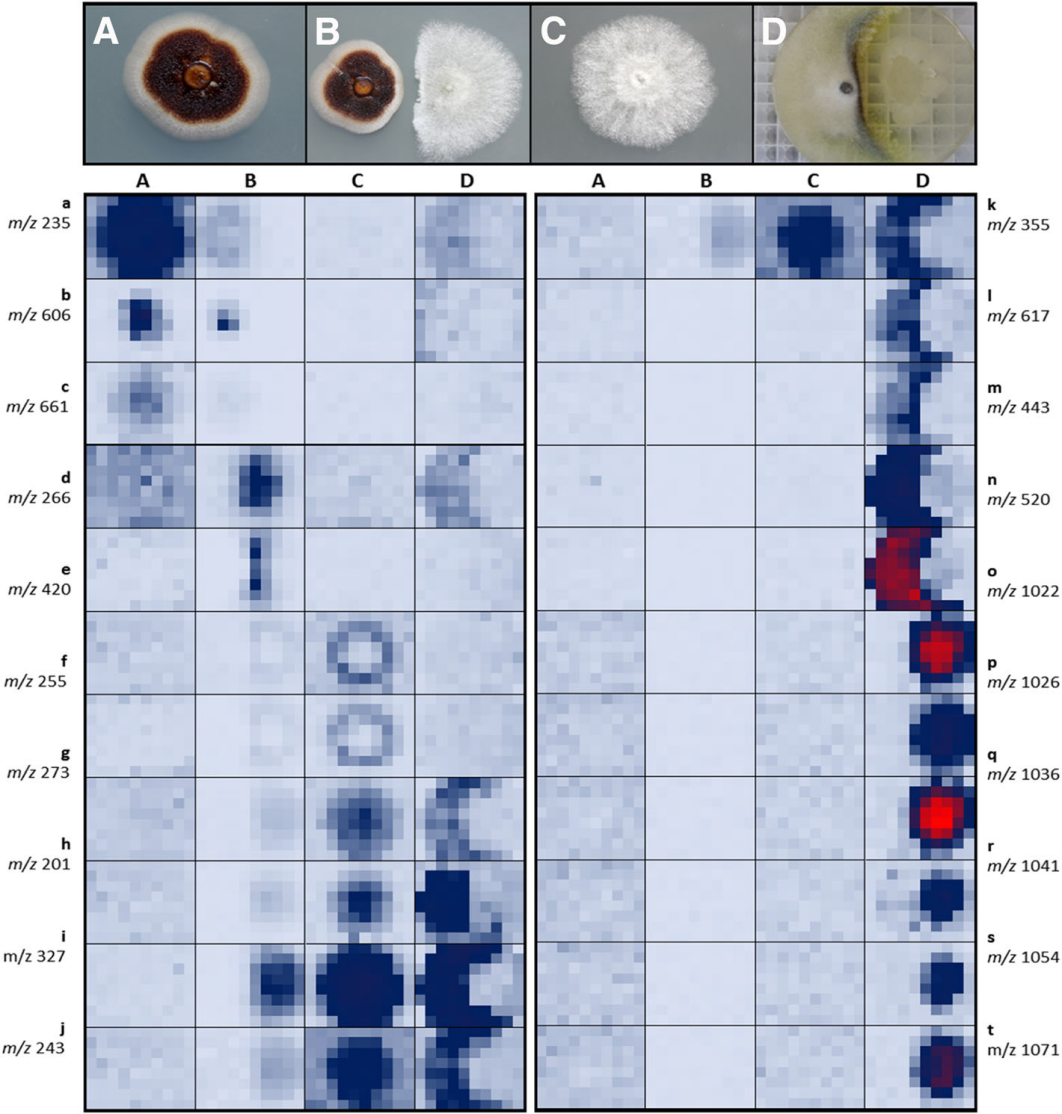
Image Mass Spectrometry (IMS) for *Dothiora* sp. (**A**), *Dothiora* sp. vs *H. mediterraneum* (**B**), *H. mediterraneum* (**C**) and *H. mediterraneum* vs *Bacillus* sp. (**D**), obtained by processing LC-MS raw data with MASS-Studio 1.0 tool. Twenty (a-t) MS components that presented differential spatial distributions depicted

Initial data management was performed with HPLC-Studio 2.0 software from UV traces of axenic and co-culture of *Dothiora* sp. vs *H. mediterraneum*. Images highlighted four components only produced when both strains interacted, apart from a large list of other compounds produced constitutively by each axenic growth (Fig. 4a-d). UV-visible detection, although sensitive enough when components present chromophores, gives very few information on the complexity of a microbial fermentation or a natural products extract. Most of natural products present 210 nm absorption relying their identification on its retention time in an HPLC chromatography and the UV-visible absorption spectrum comparing with standards. Mass spectrometry is a detection technique with the ability to discriminate molecular mases of the different components of a mixture with more sensitivity and accuracy. MASS-Studio 1.0 was adapted to generate these high detailed images of both the coculture and the individual strains (Fig. 5a-c). Results highlighted many other components produced constitutively by the strains and, more interestingly, many others that were produced only during their interaction. Moreover, spatial correlation of the antimicrobial area could be correlated with the ion with a mass to charge ratio (m/z) of 419.2421. Not present in the axenic cultures (Fig. 5B, e).

**Fig. 6:**
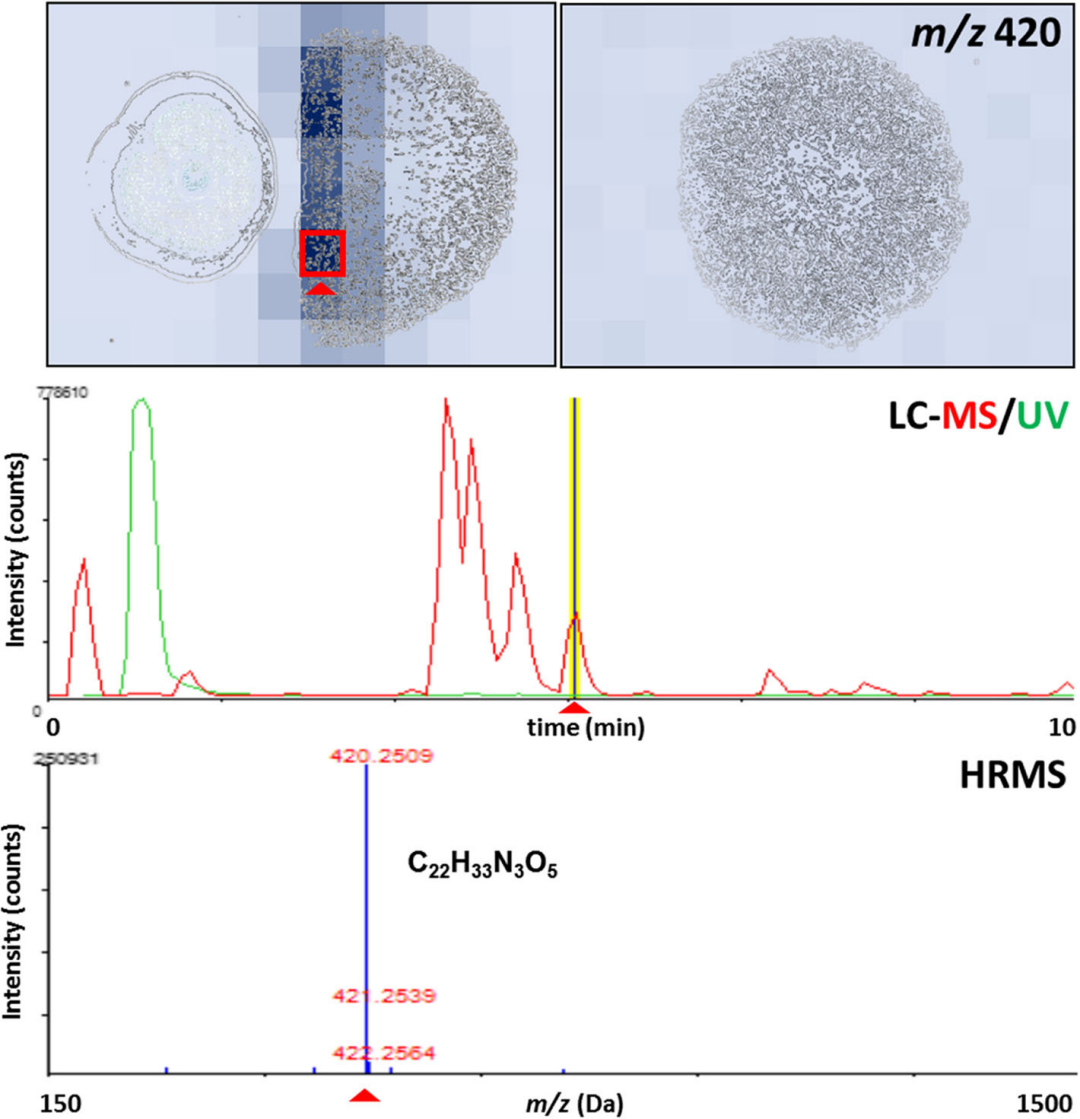
Imaging Mass Spectrometry (IMS) distribution and LC/HRMS dereplication of most induced component in the interaction of *Dothiora* sp. vs *H. mediterraneum*

The co-localization of this ion in the inhibition zone suggested that this molecule could be involved in the antagonistic activity observed. The LC-HRMS dereplication by comparison with more than 900 microbial natural product standards (60% with anti-infective activity) did not give a positive match [19]. A tentative molecular formula could be determined by BrukerTM Smart formula 3D® Software, according to its high-resolution mass isotopic pattern, as C22H33N3O5 (Fig. 6).

In the case of co-culturing *Hypoxylon mediterraneum* with *Bacillus* sp. (Fig. 5d) twelve known compounds with antimicrobial activities were dereplicated as possible responsible of part of the interactions observed. These compounds belonged to three main families produced by *Bacillus* sp.: surfactins (Fig. 5D, o and D, q), iturins (Fig. 5D, p and D, r) and mycosubtilins (Fig. 5D, s and D, t) [21, 22]. Other accumulated ions were found to be produced by *H. mediterraneum* in response to this antimicrobial attack, as (m/z) 263, 337, 429, 443, 520 or 709, but were not observed when the strain was grown axenically. This agreed with the selective production of several secondary metabolites as a signaling response or a defense mechanism.

## Conclusions

A combination of compound management techniques, automated micro-extractions and the HPLC-Studio 2.0 and MASS-Studio 1.0 software tools was used for the development of Ultra Violet and Mass Spectrometry Imaging from microbial interactions. Spatial localization of secondary metabolites resulted in an advanced tool for the evaluation of the antagonistic effects among strains in ecological communities. Mass Spectrometry Imaging (IMS) resulted in a more informative analytical technique than Ultraviolet Imaging (IUV) for the evaluation of these microbial interactions. Moreover, this methodology, when combined with evaluation of antimicrobial properties, can speed up the discovery of bioactive natural products and signaling molecules.
